# Association Between Atherogenic Index of Plasma and Clinical Outcomes in Peritoneal Dialysis Population

**DOI:** 10.3390/jcm14145030

**Published:** 2025-07-16

**Authors:** Jiayao Lan, Chunyan Yi, Ruihua Liu, Jing Guo, Shiyan Tu, Haishan Wu, Jianxiong Lin, Haiping Mao, Hongjian Ye, Wei Chen, Xiao Yang

**Affiliations:** 1 Department of Nephrology, The First Affiliated Hospital, Sun Yat-sen University, 58th Zhongshan Road II, Guangzhou 510080, China; lanjy8@mail2.sysu.edu.cn (J.L.); yichy@mail.sysu.edu.cn (C.Y.); liurh39@mail.sysu.edu.cn (R.L.); guoj235@mail2.sysu.edu.cn (J.G.); tushy@mail2.sysu.edu.cn (S.T.); wuhsh6@mail.sysu.edu.cn (H.W.); ljianx@mail.sysu.edu.cn (J.L.); maohp@mail.sysu.edu.cn (H.M.); chenwei99@mail.sysu.edu.cn (W.C.); 2 Key Laboratory of Nephrology, Ministry of Health and Guangdong Province, Guangzhou 510080, China

**Keywords:** atherogenic index of plasma, peritoneal dialysis, all-cause mortality, cardiovascular mortality, peritonitis, clinical outcomes

## Abstract

**Background**: The atherogenic index of plasma (AIP), a prognostic indicator for cardiovascular disease, has not been fully explored in relation to clinical outcomes in patients receiving peritoneal dialysis. This study aims to elucidate the relationship between baseline AIP levels and all-cause mortality, cardiovascular mortality, and the peritonitis risk in this population. **Methods**: This retrospective cohort study included incident peritoneal dialysis patients in our center from 1 January 2006 through 31 December 2021. The end of the follow-up time was 31 December 2023. The participants were stratified by baseline AIP levels. Kaplan–Meier curves, Cox regression analyses, and subgroup analyses were used to evaluate associations with clinical outcomes. **Results**: The average age of the 2460 participants in this study was 45.9 years, and 1456 (59.2%) of them were men. Diabetic nephropathy (19.5%) was the second most common kidney disease, after primary glomerulonephritis (60.8%). The higher AIP tertile group was significantly associated with increased risks of all-cause mortality, cardiovascular mortality, and peritonitis compared to the lowest AIP group, as evidenced by the Kaplan–Meier curves and the multivariate analyses. Continuous AIP levels also showed a positive correlation with the all-cause mortality and peritonitis risk, even after controlling for covariates. **Conclusions**: Our study highlights AIP as a predictive marker for adverse outcomes in PD patients, emphasizing its potential utility in risk stratification and clinical management.

## 1. Introduction

Dyslipidemias, characterized by abnormal serum levels of cholesterol, triglycerides, or both, along with associated lipoprotein abnormalities, represent a significant health concern in peritoneal dialysis (PD) patients. Compared to non-dialysis chronic kidney disease (CKD) and hemodialysis (HD) populations, PD patients exhibit a more atherogenic lipid profile. This distinction is primarily attributed to the unique metabolic challenges associated with PD, including continuous glucose absorption from dialysis solutions and peritoneal protein loss [1,2,3]. Emerging evidence suggests that dyslipidemia in PD patients is not merely a metabolic abnormality but a significant risk factor for various clinical outcomes, including treatment failure in PD-related peritonitis [4], technique failure [5], and accelerated decline in residual renal function [6]. The most frequently reported clinical consequence of dyslipidemia is the increased risk of atherosclerotic cardiovascular disease (CVD), characterized by elevated low-density lipoprotein cholesterol (LDL-C), triglycerides (TG), and lipoprotein(a) (LP (a)) and decreased high-density lipoprotein cholesterol (HDL-C) [2]. The atherogenic index of plasma (AIP), calculated as log10 (TG/HDL-C), has emerged as a valuable predictive indicator for cardiovascular risks and plaque progression [7,8]. Numerous studies have demonstrated its association with various cardiovascular outcomes, including myocardial infarction [9], ischemic stroke [10], and carotid atherosclerosis [11,12]. The relationship between AIP and mortality has been extensively studied in different populations. A nationwide population-based cohort study identified a significant association between a higher AIP and increased diabetes mortality in women over 65 years, while demonstrating a U-shaped relationship with all-cause mortality [13]. Similarly, a longitudinal investigation found that both low and high AIP levels were linked to increased all-cause mortality, with a U-shaped association observed with CVD mortality, specifically in hypertensive patients [14]. In the context of dialysis patients, research on AIP remains limited but increasingly significant. Mi Jung Lee et al. identified a U-shaped correlation between AIP and all-cause mortality in both HD and PD patients [15]. This link was further clarified by multicenter cohort research conducted in China, which found a nonlinear, U-shaped association with an identified inflection point at 0.63 and an inverse relationship between AIP and the all-cause mortality risk in the highest tertile compared to the lowest tertile group [16]. Furthermore, in patients receiving continuous hemodialysis, AIP has been linked to clinical outcomes, including intradialytic hypotension and stroke [17,18]. However, significant gaps remain in our understanding, particularly regarding the relationship between AIP and CVD mortality, as well as peritonitis in PD patients.

This research aims to address these critical knowledge gaps by investigating the associations between AIP and clinical outcomes in PD patients, including all-cause mortality, CVD mortality, and peritonitis. By exploring these relationships, we hope to provide valuable insights into the prognostic value of AIP in this vulnerable population and potentially identify new avenues for risk stratification and therapeutic intervention.

## 2. Methods

### 2.1. Population

In this retrospective cohort study, we included PD patients who had PD catheter insertions performed and underwent peritoneal dialysis treatment at our center between 1 January 2006 and 31 December 2021. The exclusion criteria were (1) individuals who were younger than 18 years old at PD initiation; (2) PD withdrawal within 6 months; (3) transition from maintenance HD to PD; (4) prior kidney transplantation before PD catheter insertion; (5) missing baseline data; and (6) unavailable baseline TG and HDL-C levels for AIP calculation. The follow-up period ended on 31 December 2023.

### 2.2. Demographic and Clinical Characteristics and Baseline Laboratory Data

At the initiation of peritoneal dialysis, we collected comprehensive baseline demographic and clinical characteristics of the enrolled individuals. These included their age, gender, body mass index (BMI), primary kidney disease, 24-h urine volume, and blood pressure; the use of angiotensin-converting enzyme inhibitors (ACEIs)), angiotensin receptor blockers (ARBs), erythropoietin (EPO), and lipid-lowering drugs; and the history of cardiovascular diseases and diabetes, including the type of dialysis and the content of the dialysis fluid. Additionally, baseline laboratory data were obtained during the first 1–3 months following PD initiation, including total cholesterol (TC), triglyceride (TG), high-density lipoprotein cholesterol (HDL-C), low-density lipoprotein cholesterol (LDL-C), hemoglobin (Hb), serum albumin (ALB), serum calcium, serum phosphorus, blood urea nitrogen (BUN), serum creatinine (Scr), blood glucose, uric acid (UA), and intact parathyroid hormone (iPTH). The baseline estimated glomerular filtration rate (eGFR) was calculated using the baseline Scr values and the Chronic Kidney Disease Epidemiology Collaboration (CKD-EPI) equation [19]. The AIP was computed using the following formula: log10 (TG/HDL-C) [7]. Based on the baseline AIP tertiles, all the individuals were categorized into distinct groups for further analysis.

### 2.3. Outcomes and Definitions

The primary outcomes of this study were all-cause mortality and CVD mortality, with PD-related peritonitis as the secondary outcome. Cardiovascular diseases (CVDs) were defined as angina pectoris, myocardial infarction, stroke, heart failure, percutaneous coronary intervention, and coronary artery bypass grafting. All-cause mortality was defined as death from any cause, regardless of etiology. Death from cardiovascular and cerebrovascular conditions, such as cardiomyopathy, cardiac arrhythmia, atherosclerotic heart disease, acute myocardial infarction, congestive heart failure, cardiac arrest, anoxic encephalopathy, ischemic brain injury, cerebrovascular disease, and peripheral arterial disease, was referred to as CVD mortality [20]. PD-related peritonitis was diagnosed based on established clinical criteria, requiring at least two of the following conditions to be present: (1) clinical signs of peritonitis, such as abdominal pain and/or cloudy dialysis effluent; (2) a dialysis effluent white cell count of >100/µL or >0.1 × 10^9^/L (after a dwell period of at least 2 h), with >50% polymorphonuclear leukocytes; (3) a positive dialysis effluent culture [21]. Censored events included transferring to other dialysis modalities, loss to follow-up before 31 January 2023, kidney transplantation, and the end of the study phase.

### 2.4. Statistical Analysis

The participants were stratified into tertiles based on baseline AIP levels: Tertile 1 (<−0.05), Tertile 2 (−0.05 to 0.20), and Tertile 3 (>0.20). Continuous variables were expressed as mean ± standard deviation for normally distributed data or median (interquartile range) for non-normally distributed data. Categorical variables were presented as frequencies (percentages). Differences between groups were analyzed using the chi-square test, Kruskal–Wallis test, or One-Way analysis of variance, as appropriate. A survival analysis was conducted using Kaplan–Meier curves with log-rank tests for hazard ratio estimation. The AIP was used as a continuous and categorical variable in Cox proportional hazards regression models to assess the relationship between the AIP and the risk of peritonitis, CVD mortality, and all-cause mortality. The unadjusted data was represented by Model 1. Age, gender, a history of cardiovascular disease, a history of diabetes mellitus, BMI, ALB, UA, calcium, LDL-C, BUN, iPTH, glucose, and hemoglobin were all taken into account when adjusting the data in Model 2. Model 3 was further adjusted for lipid-lowering medications. The results were expressed as hazard ratios (HRs) and 95% confidence intervals (CIs). We divided the patients into subgroups according to their age, gender, BMI, history of CVD, and diabetes mellitus to explore the association between the AIP and the outcomes in our study. All the statistical analyses were conducted using IBM SPSS Statistics 25.0 and R version 4.4.1, with a two-tailed *p* < 0.05 considered statistically significant.

## 3. Results

### 3.1. Study Population and Baseline Characteristics Classified by Baseline AIP

**Figure 1:**
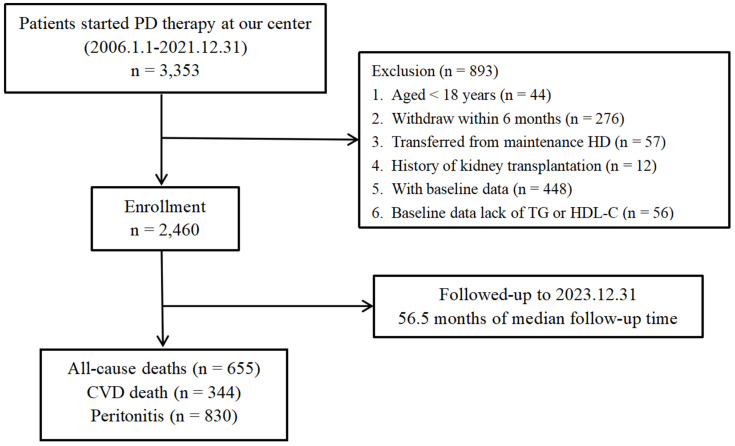
A flowchart of the study population. Abbreviations: PD, peritoneal dialysis; HD, hemodialysis; TG, triglyceride; HDL-C, high-density lipoprotein cholesterol; CVD, cardiovascular diseases.

A total of 3353 patients undergoing continuous ambulatory PD therapy at our center between 1 January 2006 and 31 December 2023 were initially included. After applying exclusion criteria, 893 patients were excluded, resulting in a final cohort of 2460 patients (Figure 1). The enrolled participants had a mean age of 45.9 ± 14.6 years, with 1456 (59.2%) being male. Following primary glomerulonephritis (60.8%), diabetic nephropathy (19.5%) ranked as the second most prevalent kidney disease. The majority of the enrolled patients chose CAPD as their dialysis method (93.1%) and used 1.5% DS (99%) for treatment. Baseline demographic and clinical characteristics were summarized in Table 1. Significant differences were observed across the three AIP groups for the history of CVD (*p* = 0.001), the use of ARBs (*p* = 0.041), SBP (*p* = 0.001), DBP, age, BMI, primary kidney diseases, diabetes history, the use of lipid-lowering drugs, AIP, and various laboratory parameters, including LDL-C, HDL-C, TG, ALB, calcium, glucose, BUN, UA, and iPTH (all *p* < 0.001) and Hb (*p* = 0.041).

### 3.2. Associations of AIP with All-Cause and CVD Mortality

**Figure 2:**
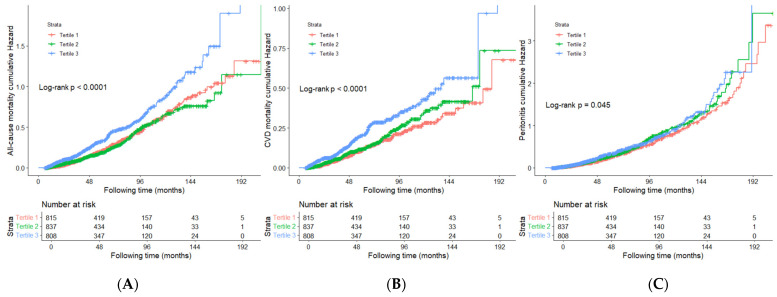
Kaplan–Meier curves for patients with different levels of AIP: all-cause mortality (**A**), cardiovascular mortality (**B**), and peritonitis (**C**).

During a median follow-up of 56.5 months, 655 all-cause mortality events and 344 CVD mortality events were recorded (Figure 1). As illustrated in Figure 2, Kaplan–Meier analyses revealed that the patients in the highest AIP tertile had significantly higher cumulative hazards for all-cause mortality (log-rank test, *p* < 0.0001) and CVD mortality (log-rank test, *p* < 0.0001) compared to the lower tertiles.

As shown in Table 2, higher AIP levels were significantly associated with an increased risk of all-cause mortality (HR: 1.814, 95% CI: 1.402–2.349, *p* < 0.001) and CVD mortality (HR: 1.892, 95% CI: 1.326–2.699, *p* < 0.001) in the unadjusted model. After adjusting for potential confounders, the association between the continuous AIP and all-cause mortality remained significant, with HRs of 1.419 (95% CI: 1.072–1.878, *p* = 0.014) in Model 2 and 1.421 (95% CI: 1.075–1.879, *p* = 0.014) in Model 3. However, the association between the AIP and CVD mortality was attenuated in adjusted models. When the AIP was presented as a categorical variable, the highest tertile (Tertile 3) was associated with significantly higher risks of all-cause mortality and CVD mortality across all the models compared with the referenced group (Tertile 1). For all-cause mortality and CVD mortality, the HRs in Model 3 were 1.280 (95% CI: 1.046–1.567, *p* = 0.017) and 1.430 (95% CI: 1.078–1.899, *p* = 0.013), respectively. The analysis results were shown in Table 2.

### 3.3. Associations of AIP and PD-Related Peritonitis in PD Patients

During follow-up, a total of 830 patients developed peritonitis. The incidence rate of peritonitis was 0.13 per person-year. As Figure 2 presented, the patients in the highest AIP tertile had significantly higher cumulative hazards for peritonitis (log-rank test, *p* = 0.045).

In the Cox regression analysis, higher continuous AIP levels were associated with an increased risk of peritonitis, with HRs of 1.298 (95% CI: 1.027–1.640, *p* = 0.029) in Model 1, 1.436 (95% CI: 1.119–1.842, *p* = 0.004) in Model 2, and 1.435 (95% CI: 1.118–1.841, *p* = 0.005) in Model 3. When the AIP was analyzed as a categorical variable, the patients in Tertile 2 had a higher risk of peritonitis compared to those in Tertile 1 in Model 2 (HR: 1.240, 95% CI: 1.048–1.466, *p* = 0.012) and Model 3 (HR: 1.232, 95% CI: 1.041–1.457, *p* = 0.015). The highest AIP tertile (Tertile 3) was associated with a significantly increased risk of peritonitis across all the models, with HRs of 1.230 (95% CI: 1.036–1.461, *p* = 0.018) in the unadjusted model, 1.309 (95% CI: 1.092–1.569, *p* = 0.004) in Model 2, and 1.304 (95% CI: 1.088–1.563, *p* = 0.004) in Model 3. The results were shown in Table 2.

### 3.4. Subgroup Analysis

**Figure 3:**
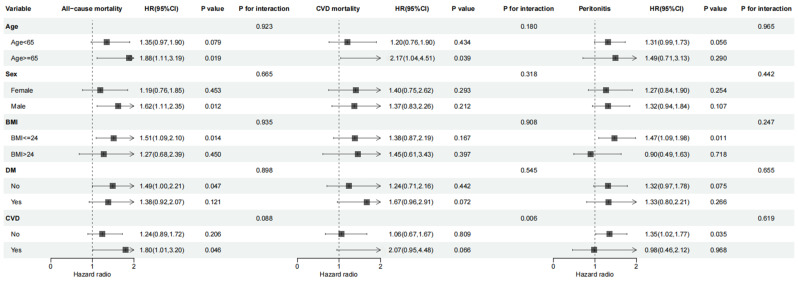
Subgroup analyses. A comparison of the adjusted hazard ratios of all-cause mortality, cardiovascular mortality, and peritonitis for the subgroups was presented by forest plot. Adjusted for age, gender, body mass index, history of diabetes mellitus, history of cardiovascular disease, serum albumin, uric acid, calcium, low-density lipoprotein cholesterol, blood urea nitrogen, intact parathyroid hormone, glucose, hemoglobin, and lipid-lowering drugs.

Subgroup analyses were performed to further explore the associations between the AIP and clinical outcomes, adjusting for confounders such as age, sex, CVD history, diabetes history, BMI, ALB, UA, calcium, LDL-C, BUN, iPTH, glucose, Hb, and lipid-lowering medication use (Figure 3). The interaction test showed that the association of the AIP with higher CVD mortality differed significantly between the patients with a CVD history and without a CVD history (*p* for interaction = 0.006). An elevated AIP was associated with a significantly higher risk of all-cause mortality in subgroups of patients aged ≥65 years (HR: 1.88, 95% CI: 1.11–3.19, *p* = 0.019), males (HR: 1.62, 95% CI: 1.11–2.35, *p* = 0.012), those with BMI ≤ 24 kg/m^2^ (HR: 1.51, 95% CI: 1.09–2.10, *p* = 0.014), and those without diabetes (HR: 1.49, 95% CI: 1.00–2.21, *p* = 0.047) or with CVD history (HR: 1.80, 95% CI: 1.01–3.20, *p* = 0.046). Among individuals aged ≥65 years, a higher AIP was linked to a 2.17-fold increased risk of cardiovascular mortality (95% CI: 1.04–4.51, *p* = 0.039). Similarly, in the patients with a BMI of ≤ 24 kg/m^2^, an elevated AIP was associated with a 1.47-times-greater likelihood of developing peritonitis (95% CI: 1.09–1.98, *p* = 0.011). However, the subgroup analyses revealed no significant interaction between AIP and either all-cause mortality or the peritonitis incidence.

## 4. Discussion

Using a retrospective design, we assessed how the AIP correlated with survival outcomes (all-cause and cardiovascular mortality) and the peritonitis risk in PD patients. Our findings indicated that elevated AIP levels were significantly associated with an increased risk of all-cause mortality, even after adjusting for potential confounders. Furthermore, the highest AIP tertile (Tertile 3) demonstrated a statistically significant increase in the risk of all-cause mortality, CVD mortality, and peritonitis compared to the lowest AIP tertile (Tertile 1). These results underscored the potential role of the AIP as a prognostic marker in PD patients.

We demonstrated a more significant association of the AIP with all-cause and CVD mortality in PD patients aged ≥65 years. Notably, the all-cause mortality risk was significantly increased in male patients, although no interaction was observed in the gender subgroup analysis. The AIP has emerged as a robust predictor of cardiovascular and cerebrovascular disease risks, surpassing traditional lipid risk factors [22,23,24,25]. Numerous studies have highlighted the association between the AIP and major adverse cardiovascular events (MACEs) across diverse populations. For instance, a large-scale analysis revealed that the risk of MACEs increased progressively with higher AIP quartiles in the Korean population [8]. Similarly, another study found that the AIP was associated with a higher risk of incident MACEs, with the association being particularly pronounced in women under 60 years of age and without hypertension or diabetes [26]. In diabetic populations, the AIP has been identified as an independent indicator of the lipid profile status, with studies, such as the ACCORD trial, linking the AIP to an increased risk of MACEs in patients with type 2 diabetes mellitus [27,28]. Additionally, research in patients undergoing percutaneous coronary intervention (PCI) has shown that lower AIP values were associated with reduced cardiovascular events post-PCI, further supporting its prognostic value [27,29]. In the research involving PD patients, a nonlinear, U-shaped relationship was found between the AIP and all-cause mortality [16]. A study involving 4403 American patients with CKD found that there was a significant linear relationship between the AIP and the risk of all-cause mortality [30]. Another study has also shown that in patients with cardiovascular–kidney–metabolic syndrome, the AIP was positively correlated with all-cause mortality and CVD mortality [31]. Gender-specific analyses have revealed intriguing differences in the relationship between the AIP and clinical outcomes. For example, a case-control study in China demonstrated that the AIP was positively associated with the risk and the severity of atherosclerotic coronary lesions in elderly male patients [32]. Conversely, in elderly women with arterial hypertension, the AIP was linked to a higher risk of all-cause mortality, independent of other risk factors [33]. A study in the Lithuanian population demonstrated that higher AIP levels were associated with increased CVD mortality in men and all-cause mortality in women [34], which differed from the results of our study. However, not all studies have corroborated these findings; for instance, the AIP was not found to be an independent risk factor for CVD in Cameroonian, postmenopausal women [35]. These discrepancies may be attributed to variations in study populations and ethnicities. Studies have shown that aging alters lipid profiles and impairs the anti-inflammatory and antioxidant functions of HDL, thereby accelerating atherosclerosis [36,37]. Numerous studies have also reported age-dependent associations between the AIP and clinical outcomes. A long-term follow-up study of elderly patients (aged >75 years) with acute coronary syndrome demonstrated that the AIP was an independent predictor of all-cause mortality in this population [38]. The results of a prospective study of middle-aged and older British adults indicated that sustained high AIP levels were linked to CVD incidence, suggesting that a persistently elevated AIP may contribute to CVD development [39]. The heterogeneity in these findings may stem from differences in study populations and age stratification, underscoring the need for larger-scale studies to clarify the prognostic value of the AIP across diverse populations.

Mechanisms linking the AIP to all-cause and CVD mortality involve atherosclerosis, insulin resistance (IR), oxidative stress, and renin–angiotensin–aldosterone system (RAAS) activation. Atherosclerosis, the most common form of cardiovascular disease [40], is a central pathway through which an elevated AIP may contribute to increased mortality. A high AIP is often associated with elevated LDL-C and reduced HDL-C levels. Specifically, a raised AIP correlates with an increase in small, dense LDL (sdLDL) particles, which more easily penetrate the arterial wall due to their smaller size. Their high affinity for proteoglycans and low affinity for LDL receptors further promote vascular deposition, accelerating the atherosclerosis [37]. Concurrently, decreased HDL-C levels impair theanti-inflammatory, antioxidant, vasodilatory, and cholesterol reverse transport functions, exacerbating atherosclerotic progression [36]. Supporting this, mass spectrometry and biochemical analyses of HDL in end-stage renal disease (ESRD) patients on hemodialysis revealed an altered HDL composition and an impaired cholesterol efflux capacity, underscoring HDL’s role in atherosclerosis in this population [41]. Additionally, elevated triglyceride levels drive the release of proinflammatory cytokines, triggering endothelial dysfunction, monocyte-derived reactive oxygen species (ROS) production, and LDL oxidation. These processes foster foam cell formation, smooth muscle cell migration, and plaque development [42]. Insulin resistance represents another critical mechanism linking AIP to CVD mortality. AIP is closely associated with IR, as excess triglycerides compete with glucose for cellular uptake, impairing glucose oxidation and promoting IR. Reduced HDL levels further diminish insulin sensitivity and β-cell function, worsening IR [43]. Moreover, IR-induced adipocyte hypertrophy recruits macrophages and other immune cells to amplify inflammation and lipid metabolic disturbances. Hyperinsulinemia associated with IR also disrupts immune cell function, shifting the balance toward proinflammatory responses and endothelial damage [44,45,46,47]. Notably, AIP has been validated as a marker of IR severity, reinforcing its role in CVD risk assessment [48,49]. A multicenter retrospective study of peritoneal dialysis patients found that IR was prevalent and correlated with an elevated risk of coronary artery disease [50]. Furthermore, oxidative stress further mediates AIP-related mortality. AIP-related dyslipidemia promotes intracellular lipid accumulation, mitochondrial dysfunction, and excessive ROS production to intensify oxidative stress and systemic inflammation [51,52]. Moreover, elevated LDL-C associated with the AIP can activate RAAS and increase angiotensin II (Ang II) activity. Ang II exacerbates endothelial dysfunction and inflammatory responses to compound the CVD risk [53]. In summary, AIP contributes to all-cause and CVD mortality through multiple interconnected mechanisms: accelerating atherosclerosis, exacerbating IR, amplifying oxidative stress, and activating RAAS. These pathways collectively underscore AIP’s value as a prognostic marker and a potential therapeutic target in high-risk populations.

PD-related peritonitis is a common complication of peritoneal dialysis, leading to the failure of peritoneal dialysis techniques and even death. According to our findings, increased AIP levels were linked to a higher risk of peritonitis among individuals receiving peritoneal dialysis. An elevated AIP may promote peritonitis through multiple mechanisms. Firstly, AIP-related dyslipidemia can induce mitochondrial dysfunction in peritoneal mesothelial cells, exacerbating oxidative stress and systemic inflammatory responses [51,52]. Secondly, an elevated AIP may promote the infiltration of inflammatory cells into peritoneal tissues, increasing the release of proinflammatory cytokines, impairing peritoneal defense mechanisms, and elevating the risk of infection [5]. Additionally, elevated LDL-C activates the RAAS, leading to endothelial dysfunction in peritoneal vessels, promoting inflammatory cell infiltration and pathogen dissemination, and aggravating oxidative stress and inflammation [53,54]. RAAS activation can also stimulate transforming growth factor-beta (TGF-β1) transcription in peritoneal mesothelial cells, inducing an epithelial- mesenchymal transition and impairing mesothelial cell function [54]. The interaction between RAAS activation and dyslipidemia further exacerbates this vicious cycle [53]. Furthermore, insulin resistance (IR) has been linked to peritonitis in PD patients [50]. Moreover, IR can contribute to the development of peritonitis by enhancing the inflammatory responses and diminishing the anti-inflammatory function of HDL [44,45,46,47]. In conclusion, an elevated AIP contributes to the peritonitis risk in PD patients via various mechanisms, such as modulating oxidative stress and inflammatory responses, stimulating the RAAS pathway, and exacerbating insulin resistance. Further studies should further elucidate the specific mechanisms of AIP in peritonitis and evaluate potential strategies for preventing or treating peritonitis by modulating lipid metabolism.

Our study also explored the role of lipid-lowering drugs, which were used by 12.8% of the participants at baseline, predominantly in those with a higher AIP. While adjustment for lipid-lowering drug use did not substantially alter the association between the AIP and outcomes (Table 2), this observation aligns with mixed evidence on statin efficacy in PD populations. For instance, the Study of Heart and Renal Protection trial subgroup analysis reported that statins did not show a significant protective effect on the risk of major atherosclerotic events in peritoneal dialysis patients, possibly due to the unique metabolic milieu of PD (e.g., persistent glucose exposure, inflammation) [55]. Notably, our lipid-lowering drug data were limited to baseline use, and future studies should evaluate longitudinal adherence and non-statin therapies in this context.

## 5. Strengths and Limitations

This investigation offers notable strengths, such as a robust sample size and an in-depth assessment of the AIP’s relationship with various clinical outcomes in peritoneal dialysis patients. To the best of our understanding, this represents the first study to examine the link between the AIP and both cardiovascular mortality and peritonitis in this patient group. Nevertheless, certain limitations must be considered. Firstly, our analysis relied solely on baseline AIP values; future studies should assess longitudinal AIP fluctuations and their clinical implications. Secondly, most of the diabetic nephropathy diagnoses were clinical rather than biopsy-proven, which may have introduced a misclassification bias. Furthermore, since our cohort consisted entirely of Chinese participants, the applicability of our results to other ethnicities may be restricted. Despite these constraints, our findings offer meaningful contributions regarding the AIP’s prognostic value in PD patients and emphasize the necessity of further research to clarify its mechanistic pathways and therapeutic relevance.

## 6. Conclusions

In this extensive PD patient cohort, elevated baseline AIP levels were independently linked to higher risks of all-cause mortality, cardiovascular death, and peritonitis. These results suggest that the AIP may serve as a valuable prognostic indicator in PD patients and reinforce the critical role of lipid regulation in this population.

## Figures and Tables

**Table 1:** Baseline demographics and clinical characteristics and laboratory data of subjects, according to AIP tertile group.

Variables	Total (*n* = 2460)	AIP			*p*
		<−0.05	−0.05–0.2	>0.20	
		(*n* = 815)	(*n* = 837)	(*n* = 808)	
Age (yr)	45.9 ± 14.6	45.2 ± 14.3	44.4 ± 14.2	48.1 ± 14.9	<0.001
Male (n, %)	1456 (59.2)	473 (58.0)	486 (58.1)	497 (61.5)	0.261
BMI (kg/m^2^)	21.79 ± 3.92	20.98 ± 4.90	21.58 ± 3.17	22.81 ± 3.29	<0.001
Primary kidney disease (n, %)					<0.001
Glomerulonephritis	1496 (60.8)	531 (65.2)	528 (63.1)	437 (54.1)	
Diabetic nephropathy	479 (19.5)	139 (17.1)	146 (17.4)	194 (24.0)	
Renal vascular diseases	204 (8.3)	55 (6.7)	69 (8.2)	80 (9.9)	
^a^ Other	281 (11.4)	90 (11.0)	94 (11.2)	97 (12.0)	
Comorbidities					
Diabetes (n, %)	498 (20.2)	142 (17.4)	150 (17.9)	206 (25.5)	<0.001
CVD (n, %)	290 (11.8)	80 (9.8)	87 (10.4)	123 (15.2)	0.001
Medication use					
ACEI (n, %)	277 (11.3)	95 (11.7)	81 (9.7)	101 (12.5)	0.176
ARB (n, %)	1047 (42.6)	376 (46.1)	340 (40.6)	331 (41.0)	0.041
Lipid-lowering drugs	315 (12.8)	79 (9.7)	104 (12.4)	132 (16.3)	<0.001
EPO	1921 (78.1)	647 (79.4)	648 (77.4)	626 (77.5)	0.549
Type of dialysis					
CAPD	2291 (93.1)	764 (93.7)	771 (92.1)	756 (93.6)	0.356
APD	18 (0.7)	4 (0.5)	3 (0.4)	11 (1.4)	0.036
Content of dialysis fluid					
1.5%DS	2353 (99.0)	780 (99.1)	798 (99.0)	775 (99.0)	0.961
2.5%DS	756 (31.8)	287 (36.5)	236 (29.3)	233 (29.8)	0.003
4.25%DS	22 (0.9)	5 (0.6)	6 (0.7)	11 (1.4)	0.226
AIP	0.07 (−0.11, 0.28)	−0.20 (−0.31, −0.11)	0.07 (0.01, 0.14)	0.37 (0.28, 0.52)	<0.001
DBP (mmHg)	134 ± 19	137 ± 19	133 ± 18	133 ± 20	<0.001
SBP (mmHg)	85 ± 14	86 ± 14	86 ± 14	84 ± 14	0.001
HDL-C (mmol/L)	1.27 ± 0.40	1.58 ± 0.42	1.24 ± 0.28	0.98 ± 0.23	<0.001
LDL-C (mmol/L)	3.00 ± 0.94	2.94 ± 0.89	3.10 ± 0.95	2.96 ± 0.97	<0.001
TC (mmol/L)	5.07 ± 1.31	5.03 ± 1.25	5.05 ± 1.31	5.13 ± 1.36	0.306
TG (mmol/L)	1.43 (1.04, 2.02)	0.92 (0.74, 1.11)	1.43 (1.25, 1.69)	2.36 (1.92, 3.18)	<0.001
Hb (g/L)	107 ± 19	106 ± 19	108 ± 19	107 ± 19	0.041
ALB (g/L)	37.0 ± 4.9	36.1 ± 4.7	37.1 ± 4.9	37.6 ± 4.9	<0.001
Calcium (mmol/L)	2.25 ± 0.20	2.21 ± 0.20	2.25 ± 0.19	2.28 ± 0.21	<0.001
Phosphorus (mmol/L)	1.38 ± 0.44	1.39 ± 0.39	1.38 ± 0.42	1.39 ± 0.50	0.873
24-h urine volume (mL)	1000 (500, 1500)	950 (500, 1400)	1000 (500, 1500)	950 (500, 1500)	0.208
Scr (μmol/L)	739 ± 249	737 ± 246	740 ± 247	739 ± 255	0.970
BUN (mmol/L)	16.2 ± 5.6	17.0 ± 5.9	16.2 ± 5.3	15.5 ± 5.5	<0.001
Glucose (mmol/L)	5.57 ± 2.42	5.38 ± 2.39	5.48 ± 2.33	5.84 ± 2.52	<0.001
UA (μmol/L)	408 ± 93	393 ± 88	411 ± 91	419 ± 99	<0.001
iPTH (pg/mL)	243.6 (121.7, 400.1)	271.1 (133.7, 432.1.6)	246.8 (130.4, 398.0)	215.8 (108.2, 379.4)	<0.001
eGFR (mL/min/1.73 m^2^)	7.02 ± 2.76	7.00 ± 2.71	7.05 ± 2.81	7.01 ± 2.75	0.935

Note: Continuous variables are presented as mean ± standard deviation or median (interquartile range), and categorical variables are presented as frequency (percentage). ^a^ Others: includes chronic tubulointerstitial diseases, obstructive nephropathy, lupus nephritis, polycystic kidney diseases, and unknown. Abbreviations: ACEIs, angiotensin-converting enzyme inhibitors; AIP, atherogenic index of plasma; ALB, serum albumin; APD, automated peritoneal dialysis; ARBs, angiotensin receptor blockers; BMI, body mass index; BUN, blood urea nitrogen; CAPD, continuous ambulatory peritoneal dialysis; CVD, cardiovascular disease; DBP, diastolic blood pressure; DS, dextrose solution peritoneal dialysis fluid; eGFR, estimated glomerular filtration rate; EPO, erythropoietin; HDL-C, high-density lipoprotein cholesterol; iPTH, intact parathyroid hormone; LDL-C, low-density lipoprotein cholesterol; SBP, systolic blood pressure; Scr, serum creatinine; TC, total cholesterol; TG, triglyceride; UA, uric acid.

**Table 2:** Uni- and multivariate Cox regression analysis of outcomes and AIP.

Outcomes	Event	Model 1		Model 2		Model 3	
		HR (95% CI)	*p*	HR (95% CI)	*p*	HR (95% CI)	*p*
All-cause mortality							
Continuous AIP	655/2460	1.814 (1.402, 2.349)	<0.001	1.419 (1.072, 1.878)	0.014	1.421 (1.075, 1.879)	0.014
AIP categories							
Tertile 1	206/815	[Reference]		[Reference]		[Reference]	
Tertile 2	196/837	0.964 (0.792, 1.173)	0.715	0.953 (0.773, 1.176)	0.656	0.969(0.785,1.196)	0.768
Tertile 3	253/808	1.505 (1.251, 1.810)	<0.001	1.271 (1.039, 1.555)	0.020	1.280 (1.046, 1.567)	0.017
Cardiovascular mortality							
Continuous AIP	344/2460	1.892 (1.326, 2.699)	<0.001	1.385 (0.944, 2.033)	0.096	1.385 (0.943, 2.033)	0.096
AIP categories							
Tertile 1	96/815	[Reference]		[Reference]		[Reference]	
Tertile 2	110/837	1.165 (0.885, 1.533)	0.275	1.153 (0.862, 1.543)	0.338	1.149 (0.858, 1.539)	0.352
Tertile 3	138/808	1.754 (1.350, 2.278)	<0.001	1.433 (1.080, 1.902)	0.013	1.430 (1.078, 1.899)	0.013
Peritonitis							
Continuous AIP	830/2460	1.298 (1.027, 1.640)	0.029	1.436 (1.119, 1.842)	0.004	1.435 (1.118, 1.841)	0.005
AIP categories							
Tertile 1	271/815	[Reference]		[Reference]		[Reference]	
Tertile 2	301/837	1.168 (0.991, 1.378)	0.064	1.240 (1.048, 1.466)	0.012	1.232 (1.041, 1.457)	0.015
Tertile 3	258/808	1.230 (1.036, 1.461)	0.018	1.309 (1.092, 1.569)	0.004	1.304 (1.088, 1.563)	0.004

Model 1, unadjusted. Model 2, adjusted for age, gender, body mass index, history of diabetes mellitus, history of cardiovascular disease, serum albumin, uric acid, calcium, low-density lipoprotein cholesterol, blood urea nitrogen, intact parathyroid hormone, glucose, and hemoglobin. Model 3, adjusted for Model 2 covariates and lipid-lowering drugs.

## Data Availability

The datasets used and analyzed during the current study are available from the corresponding author on reasonable request.

## Abbreviations

ACEIs	angiotensin-converting enzyme inhibitors;
AIP	atherogenic index of plasma;
ALB	serum albumin;
Ang II	angiotensin II;
APD	automated peritoneal dialysis;
ARBs	angiotensin receptor blockers;
BMI	body mass index;
BUN	blood urea nitrogen;
CAPD	continuous ambulatory peritoneal dialysis;
CKD	chronic kidney diseases;
CVD	cardiovascular disease;
DBP	diastolic blood pressure;
DS	dextrose solution peritoneal dialysis fluid;
eGFR	estimated glomerular filtration rate;
EPO	erythropoietin;
ESRD	end-stage renal disease;
Hb	hemoglobin;
HD	hemodialysis;
HDL-C	high-density lipoprotein cholesterol;
iPTH	intact parathyroid hormone;
IR	insulin resistance;
LDL-C	low-density lipoprotein cholesterol;
MACEs	major adverse cardiovascular events;
PCI	percutaneous coronary intervention;
PD	peritoneal dialysis;
RAAS	renin–angiotensin–aldosterone system;
ROS	reactive oxygen species;
SBP	systolic blood pressure;
Scr	serum creatinine;
SdLDL	small and dense low-density lipoprotein;
TC	total cholesterol;
TG	triglyceride;
TGF-β1	transforming growth factor-beta;
UA	uric acid.
